# Supplementary material for: Symmetries in quantum networks lead to no-go theorems for entanglement distribution and to verification techniques
Source: Nat Commun. 2022 Jan 25;13:496. doi: 10.1038/s41467-022-28006-3 (PMC8789828; doi:10.1038/s41467-022-28006-3)
Supplement: Supplementary file 1 — Supplementary Information [file 41467_2022_28006_MOESM1_ESM.pdf]

Supplementary Information for 'Symmetries in quantum networks lead to no-go theorems for entanglement distribution and to verification techniques'

Kiara Hansenne, Zhen-Peng Xu, Tristan Kraft, and Otfried Gühne

SUPPLEMENTARY NOTE 1: NETWORK CORRELATIONS AND THE INFLATION TECHNIQUE

Before explaining the inflation technique in some detail, it is useful to note some basic observations on the definition of network correlations. Recall from the main text that triangle network states are of the form

$$\varrho = \sum_{\lambda} p_\lambda \mathcal{E}_A^{(\lambda)} \otimes \mathcal{E}_B^{(\lambda)} \otimes \mathcal{E}_C^{(\lambda)} \left[\varrho_{ABC}\right], \tag{S1}$$

i.e. there exist source states ϱ_a, ϱ_b, ϱ_c with $\varrho_{ABC} = \varrho_a \otimes \varrho_b \otimes \varrho_c$, a shared random variable λ and channels (that is, trace preserving positive maps) $\mathcal{E}_A^{(\lambda)}$, $\mathcal{E}_B^{(\lambda)}$ and $\mathcal{E}_C^{(\lambda)}$ that can be used to generate the state ϱ, as shown in Supplementary Fig. 1.

First, we note that in this definition, the state ϱ_{ABC} does not depend on the classical variable λ. This is however, no restriction, as the dimensions of the source states are not bounded. If in Eq. (S1) the $\varrho_{ABC}(\lambda)$ and hence the $\varrho_a(\lambda)$, $\varrho_b(\lambda)$, $\varrho_c(\lambda)$ depend on λ, one can just combine the set of all $\varrho_a(\lambda)$ to a single ϱ_a etc. and redefine the maps $\mathcal{E}_A^{(\lambda)}$ etc. such that they act on the appropriate $\varrho_a(\lambda)$. This results in a form where ϱ_{ABC} does not depend on λ anymore, hence the state can be written as in Eq. (S1). Note that this has already been observed in [1].

As mentioned in the main text, one may define network states also in a manner where the shared randomness is carried by the sources only. Indeed, if one adds an ancilla system to the source states, this may be used to identify the channel $\mathcal{E}_X$ to be applied. More explicitly, the source states may be redefined as $\varrho_c^{(\lambda)} \otimes |\lambda\rangle\langle\lambda|$ with orthogonal ancilla states $|\lambda\rangle$ being send to Bob (respectively to Charlie and Alice for sources a and b), such that Bob can, by measuring $|\lambda\rangle$, decide which channel to apply. This measurement can then be seen as a global channel $\mathcal{E}_B$ that does not depend on λ. From the linearity of the maps, one may also write general network states as $\varrho = \mathcal{E}_A \otimes \mathcal{E}_B \otimes \mathcal{E}_C \left[\sum_\lambda p_\lambda \varrho_{ABC}^{(\lambda)}\right]$ as an equivalent definition. For our purpose, the potential dependence of ϱ_{ABC} on λ has the following consequence: If we wish to compute for a symmetric $|\psi\rangle$ the maximum fidelity $\langle\psi|\varrho|\psi\rangle$ over all network states ϱ, then we may assume that ϱ permutationally symmetric, too. This follows from the simple fact that we can, without decreasing the overlap, symmetrize the state ϱ_{ABC}, and the symmetrized state will still be preparable in the network.

Second, one may restrict the $\varrho_{ABC} = \varrho_a \otimes \varrho_b \otimes \varrho_c$ further. Indeed it is straightforward to see that $\varrho_a = |a\rangle\langle a|$, $\varrho_b = |b\rangle\langle b|$, and $\varrho_c = |c\rangle\langle c|$ can be chosen to be pure, as the channels $\mathcal{E}_A^{(\lambda)}$ etc. are linear.

Third, as the set of network preparable states is by definition convex, one may ask for its extremal points. Formally, these are of the form $\mathcal{E}_A^{(\lambda)} \otimes \mathcal{E}_B^{(\lambda)} \otimes \mathcal{E}_C^{(\lambda)} \left[|a\rangle\langle a| \otimes |b\rangle\langle b| \otimes |c\rangle\langle c|\right]$, but can these further be characterized? Clearly, pure

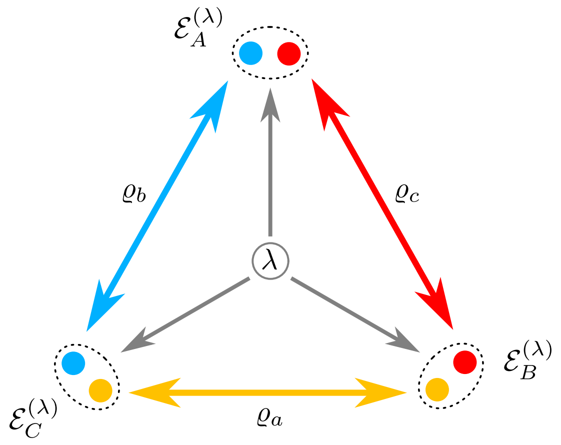

Supplementary Fig. 1: *Triangle quantum network.* Three sources ϱ_a, ϱ_b and ϱ_c distribute parties to three nodes, Alice, Bob and Charlie (A, B and C). Alice, Bob and Charlie each end up with a bipartite system $X = X_1X_2$ on which they perform a local channel $\mathcal{E}_X^{(\lambda)}$ ($X = A, B, C$) depending on a classical random variable λ.

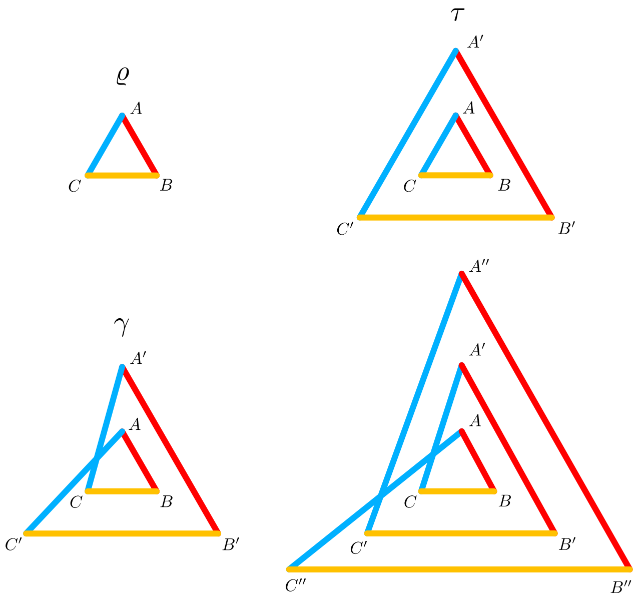

Supplementary Fig. 2: *Triangle network and three of its inflations.* The first figure represents the triangle network Supplementary Fig. 1, with global state ϱ. Using the same source states (represented by lines of same colour) and same local channels, one can build the so-called inflated state τ, which is biseparable. The state γ is build similarly, but with a different rewirering, leading to an inflated state that is in general not separable and different from τ. One may also go to higher order inflations, e.g. with three copies and some rewireing, as depicted here. This procedure implies several equalities between the marginals of the original state and its inflations.

biseparable three-particle states, such as $|\psi\rangle = |\phi\rangle_{AB} \otimes |\eta\rangle_C$ are extremal points. There are however, also mixed states as extremal points, which can be seen as follows: It was shown in Ref. [2] that pure three-qubit states which are genuine multiparticle entangled (that is, not biseparable) cannot be prepared in the triangle network. On the other hand, in Ref. [1] it was shown that there are network states having a GHZ fidelity of 0.5170, which implies that they are genuine multiparticle entangled [3]. So, the set defined in Eq. (S1) must have some extremal points, which are genuine multiparticle entangled mixed states.

After this prelude, let us explain the inflation technique [4, 5], which has already proven to be useful for the characterization of quantum networks [1]. We introduce it for triangle networks as for arbitrary networks it is a direct generalization.

We start with constructing two inflations of the triangle network. Consider two networks with six vertices and six edges as in Supplementary Fig. 2. Identical sources are distributed along the lines of same colour, thus two copies of each source are needed per network. In other words, the source ϱ_b is distributed between AC and $A'C'$ to generated τ, and between AC' and $A'C$ for γ (analogously for ϱ_a and ϱ_c, following Supplementary Fig. 2). Then, the channels are performed according to the random parameter λ. Both on primed and non-primed A nodes, the same channel $\mathcal{E}_A^{(\lambda)}$ is applied and similarly for B and C. This leaves us with two network states, τ and γ. Those operators are physical states, i.e. they have a unit trace and are positive semi-definite. Formally, they can be written as

$$\tau = \sum_\lambda p_\lambda \left(\mathcal{E}_A^{(\lambda)} \otimes \mathcal{E}_B^{(\lambda)} \otimes \mathcal{E}_C^{(\lambda)} \left[\varrho_{ABC}\right]\right) \otimes \left(\mathcal{E}_{A'}^{(\lambda)} \otimes \mathcal{E}_{B'}^{(\lambda)} \otimes \mathcal{E}_{C'}^{(\lambda)} \left[\varrho_{A'B'C'}\right]\right) \tag{S2}$$

and

$$\gamma = \sum_\lambda p_\lambda \mathcal{E}_A^{(\lambda)} \otimes \mathcal{E}_B^{(\lambda)} \otimes \mathcal{E}_C^{(\lambda)} \otimes \mathcal{E}_{A'}^{(\lambda)} \otimes \mathcal{E}_{B'}^{(\lambda)} \otimes \mathcal{E}_{C'}^{(\lambda)} \left[\varrho_{ABCA'B'C'}\right], \tag{S3}$$

where $\varrho_{ABCA'B'C'} = \varrho_c \otimes \varrho_b \otimes \varrho_a \otimes \varrho_c \otimes \varrho_b \otimes \varrho_a$, with the ordering of parties being different on both sides. Here, one needs to carefully pay attention to which channel acts on which party (this is depicted in Supplementary Fig. 2). Clearly, given only the knowledge of ϱ, the precise form of τ and γ is not known. Still, due to the way they are

constructed some of their marginals have to be equal, namely

$$\tau_{ABC} = \tau_{A'B'C'} = \varrho, \tag{S4}$$
$$\gamma_{ABC} = \gamma_{A'B'C'}, \tag{S5}$$
$$\mathrm{Tr}_{XX'}(\tau) = \mathrm{Tr}_{XX'}(\gamma) \quad \text{for} \quad X = A, B, C. \tag{S6}$$

Furthermore, from Eq. (S2) it is clear that τ is separable wrt the partition $ABC|A'B'C'$ and we note that τ and γ are permutationally symmetric under the exchange of non primed and primed vertices. Therefore, if, for some given state ϱ, it is not possible to find states τ and γ that satisfy those conditions, then ϱ cannot be generated in the considered network.

An interesting point is that the question for the existence of τ and γ with the desired properties can be directly formulated as a semidefinite program (SDP). This can be used to prove that such inflations do not exist, and the corresponding dual program can deliver an witness-like construction that can be used to exclude preparability of a state in the network. Still, these approaches are memory intensive. For instance, as the authors of Ref. [1] acknowledge, it is difficult to derive tests for tripartite qutrit states in a normal computer.

Finally, let us note that other triangle inflations may be considered, for instance inflations with $3n$ nodes ($n = 3, 4, \dots$) or simply wired differently than τ and γ. As mentioned previously, this technique can also be used for more complicated networks.

SUPPLEMENTARY NOTE 2: FIDELITY ESTIMATE FOR THE GHZ STATE

Let us compute a bound on the fidelity of triangle network states to the GHZ state, i.e. compute $F = \max\langle GHZ|\varrho|GHZ\rangle$, where the maximum is taken over all states as in Eq. (S1). For this maximization is it sufficient to consider the extremal states, which are of the type $\varrho_{ITN} = \mathcal{E}_A \otimes \mathcal{E}_B \otimes \mathcal{E}_C\left[\varrho_{ABC}\right]$, here ITN stands for the independent triangle network, that is the triangle network without shared randomness.

Therefore, one may use techniques based on covariance matrices [6, 7], which are designed for the ITN. The covariance matrix (CM) Γ of some random variables $x_1, \dots, x_N$ is the matrix with elements $\Gamma_{ij} = \mathrm{cov}(x_i, x_j) = \langle x_i x_j\rangle - \langle x_i\rangle \langle x_j\rangle$, $i = 1, \dots, N$.

We can now explain the general idea of the technique in Ref. [7]: If one computes the CM of the outcomes of Z-measurements on each qubit of an ITN state, then this matrix has a certain block structure. Checking this block structure can be done by checking the positivity of the comparison matrix $M(\Gamma)$. The comparison matrix obtained by flipping the signs of the off-diagonal elements of Γ. The condition then reads: For any quantum state in the ITN the comparison matrix of the CM is positive semi-definite. Hence, a negative eigenvalue in the comparison matrix excludes a state of being preparable in the ITN.

Now, if we apply that to states $\varrho(F) = F|GHZ\rangle\langle GHZ| + (1-F)\tilde{\varrho}$ with a fidelity F to the GHZ state, the comparison matrix of the CM reads

$$M(\Gamma) = \begin{bmatrix} 1 - a^2(1-F)^2 & -\left(F + d(1-F) - ab(1-F)^2\right) & -\left(F + e(1-F) - ac(1-F)^2\right) \\ -\left(F + d(1-F) - ab(1-F)^2\right) & 1 - b^2(1-F)^2 & -\left(F + f(1-F) - bc(1-F)^2\right) \\ -\left(F + e(1-F) - ac(1-F)^2\right) & -\left(F + f(1-F) - bc(1-F)^2\right) & 1 - c^2(1-F)^2 \end{bmatrix}, \tag{S7}$$

where $a = \langle Z11\rangle_{\tilde{\varrho}}$, $b = \langle 1Z1\rangle_{\tilde{\varrho}}$, $c = \langle 11Z\rangle_{\tilde{\varrho}}$, $d = \langle ZZ1\rangle_{\tilde{\varrho}}$, $e = \langle Z1Z\rangle_{\tilde{\varrho}}$ and $f = \langle 1ZZ\rangle_{\tilde{\varrho}}$. From the last paragraph, we have that this matrix is positive semi-definite for ITN states, thus $\langle\phi|M(\Gamma)|\phi\rangle \geq 0$, for all vectors $|\phi\rangle$, and in particular for $|\phi\rangle = (1, 1, 1)/\sqrt{3}$. We notice that $\langle\phi|M(\Gamma)|\phi\rangle$ is upper bounded by $4 - 6F + F^2$ and therefore, $0 \leq \langle\phi|M(\Gamma)|\phi\rangle \leq 4-6F+F^2$ holds for ITN states and we are able to exclude all states $\varrho(F)$ with $F > 3-\sqrt{5} \simeq 0.7639$ form the triangle network scenario.

By making use of additional constraints or other criteria, we can obtain tighter bound. For any given three compatible dichotomic measurements M_1, M_2, M_3, we have [1]

$$p(M_1 = M_2) \geq p(M_1 = M_3) + p(M_2 = M_3) - 1. \tag{S8}$$

This implies

$$\langle M_1 M_2\rangle \geq \langle M_1 M_3\rangle + \langle M_2 M_3\rangle - 1. \tag{S9}$$

By substituting M_i with $-M_i$, we obtain

$$\langle M_1M_2\rangle \geq |\langle M_1M_3\rangle + \langle M_2M_3\rangle| - 1, \tag{S10}$$

$$\langle M_1M_2\rangle \leq 1 - |\langle M_1M_3\rangle - \langle M_2M_3\rangle|. \tag{S11}$$

In our case, we have

$$d \geq |a+b| - 1, \quad e \geq |a+c| - 1, \quad f \geq |b+c| - 1. \tag{S12}$$

With this extra constraint, $0 \leq \langle\phi|M(\Gamma)|\phi\rangle \leq 9 - 12F$ holds for ITN states and we are able to exclude all states $\varrho(F)$ with $F > 3/4 = 0.75$ form the triangle network scenario.

Another criterion for ITN states [8] states that

$$\begin{aligned} &(1+|E_A|+|E_B|+E_{AB})^2 \\ +&(1+|E_A|+|E_C|+E_{AC})^2 \\ +&(1+|E_B|+|E_C|+E_{BC})^2 \\ \leq& 6(1+|E_A|)(1+|E_B|)(1+|E_C|), \end{aligned} \tag{S13}$$

where

$$E_A = \langle Z11\rangle_\varrho,\ E_B = \langle 1Z1\rangle_\varrho,\ E_C = \langle 11Z\rangle_\varrho, \tag{S14}$$

$$E_{AB} = \langle ZZ1\rangle_\varrho,\ E_{AC} = \langle Z1Z\rangle_\varrho,\ E_{BC} = \langle 1ZZ\rangle_\varrho. \tag{S15}$$

As it turns out, if Eq. (S13) together with Eq. (S12) has a feasible solution of $a, b, c, d, e, f \in [-1, 1]$, then F should be no more than $1/\sqrt{2} \simeq 0.7071$. Hence, we can exclude all states $\varrho(F)$ with $F > 1/\sqrt{2} \simeq 0.7071$ form the triangle network scenario.

In particular, if we know $E_A = E_B = E_C = 0$, i.e., $a = b = c = 0$, Eq. (S13) reduces to

$$\begin{aligned} 6 \geq\ & (1+F+d(1-F))^2 + (1+F+e(1-F))^2 \\ & + (1+F+f(1-F))^2 \\ \geq\ & 3(1+F-(1-F))^2 \\ =\ & 12F^2, \end{aligned} \tag{S16}$$

which implies $F \leq 1/\sqrt{2}$. The bound $1/\sqrt{2} \simeq 0.7071$ is slightly worse, but close to the one 0.6803 obtained in Ref. [1] based on advanced numerical computations.

SUPPLEMENTARY NOTE 3: GRAPH AND CLUSTER STATES

In this Supplementary Note we present our results on graph states and cluster states. It is structured as follows: We first recall the basic facts about graph and cluster states. Then, we prove the estimate on the fidelity with cluster states for states in the square network (see the main text). Finally, we present the proof and discussion of Observation 1.

Graph states and the stabilizer formalism

Graph states [9, 10] are quantum states defined through a graph $G = (V, E)$, i.e. through a set V of N vertices and a set E containing edges that connect the vertices. The vertices represent the physical systems, qubits. One way of describing these states is through the stabilizer formalism. For that, as introduced in the main text, one first needs to introduce the generator operators g_i of graph states: a graph state $|G\rangle$ is the unique common +1-eigenstate of the set of operators $\{g_i\}$,

$$g_i = X_i \prod_{j\in\mathcal{N}_i} Z_j, \tag{S17}$$

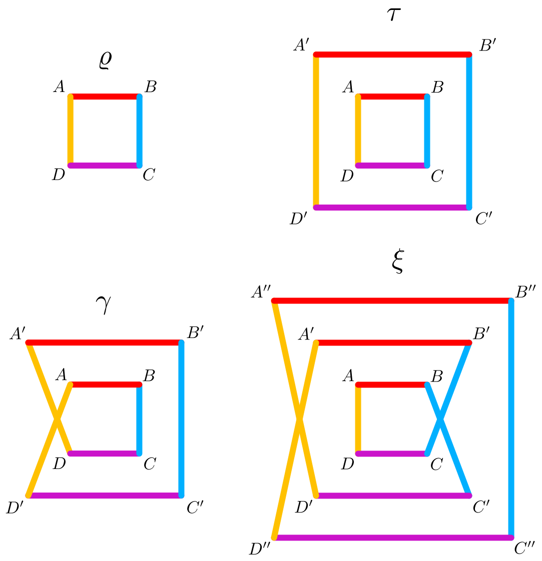

Supplementary Fig. 3: *Square network and three of its inflations.* Similar to the triangle network of Supplementary Fig. 2, the states τ and γ are generated using two copies of the sources and channels used to generate ϱ, without and with rewireing respectively. Then, one goes to a high order inflation by using three copies of the sources and the local channels. By rewireing according to the figure, one gets the inflated state ξ. Due to the way network states are generated, several of the marginals of ϱ, τ, γ and ξ are identical.

where $\mathcal{N}_i$ is the neighbourhood of the qubit i, i.e. the set of all qubits $j \in V$ connected to the qubit $i \in G$. The state $|G\rangle$ can also be described through its stabilizer, which is the set $\mathcal{S} = \{S_1, \ldots, S_{2^N}\} = \{\prod_{i=1}^N g_i^{x_i} : \{x_1, \ldots, x_N\} \in \{0,1\}^N\}$. This means that $\mathcal{S}$ contains all possible products of the generators g_i, hence $S_i|G\rangle = |G\rangle$. We note that $\mathbb{1} \in \mathcal{S}$. The projector onto the state $|G\rangle$ reads

$$|G\rangle\langle G| = \frac{1}{2^N} \sum_{i=1}^{2^N} S_i. \tag{S18}$$

Defined like that, graph states are a subset of the more general stabilizer states [9, 11, 12]. First, one has to consider an abelian subgroup $\mathcal{S}$ of the Pauli group $\mathcal{P}_N$ on N qubits that does not contain the operator $-\mathbb{1}$. To that set corresponds a vector space $V_{\mathcal{S}}$ that is said to be stabilized by $\mathcal{S}$, i.e. every element of this vector space is stable under the action of any element of $\mathcal{S}$. We call stabilizers that lead only to one state full-rank stabilizers, i.e. there is a unique common eigenstate with eigenvalue $+1$. That state is completely determined by a subset of N elements of $\mathcal{S}$. As an example, one may consider the GHZ state, as explained in the main text. Indeed, it is the unique common eigenstate of XXX, $1ZZ$ and $ZZ1$. One can show that any stabilizer state is, after a suitable local unitary transformation, equivalent to a graph state.

More precisely, the local unitary transformations that map any stabilizer state to a graph state belong to the so-called *local Clifford group* $\mathcal{C}_1$. The local Clifford group is defined as the normalizer of the single-qubit Pauli group, i.e. $U\mathcal{P}_1U^\dagger = \mathcal{P}_1$ for all $U \in \mathcal{C}_1$. By construction, the stabilizer formalism is preserved under the action of the local Clifford group, and hence, an interesting question is under which conditions two graph states (or two stabilizer states) are equivalent under local Cliffords. For graph states this question has a simple solution in terms of graphical operations that determine their equivalence. Namely, two graph states are equivalent under the action of the local Clifford group if and only if their corresponding graphs are equivalent under a sequence of *local complementations* [13]. For a given graph G and vertex $i \in V$ the local complement G' of G at the vertex i is constructed in two steps. First, we have to determine the neighborhood $N(i) \subset V$ of the vertex i and then the induced subgraph is inverted, i.e. considering all possible edges in the neighborhood any pre-existing edge is removed and any non-existing edge is added. E.g. having a graph $V = \{1, 2, 3\}$ and $E = \{(1,2), (2,3), (3,1)\}$, a local complementation on vertex 1 results in the graph with edges $E = \{(1,2), (3,1)\}$.

Stabilizer elements				
$S_1 = XZ1Z$	$S_5 = YYZZ$	$S_9 = X1X1$	$S_{11} = -YXY1$	$S_{15} = XXXX$
$S_2 = ZXZ1$	$S_6 = YZZY$	$S_{10} = 1X1X$	$S_{12} = -1YXY$	
$S_3 = 1ZXZ$	$S_7 = ZYYZ$		$S_{13} = -Y1YX$	
$S_4 = Z1ZX$	$S_8 = ZZYY$		$S_{14} = -XY1Y$	
$\langle\cdot\rangle_\varrho = \Theta$	$\langle\cdot\rangle_\varrho = \Lambda$	$\langle\cdot\rangle_\varrho = \Xi$	$\langle\cdot\rangle_\varrho = -\Sigma$	$\langle\cdot\rangle_\varrho = \Omega$

Supplementary Table I: Elements of the stabilizer of the four-qubit cluster states. The qubit indices A, B, C, D are suppressed here. See the text for further details.

Estimate for the cluster state fidelity

We aim at computing a bound on the fidelity of square network states to the four-qubit ring cluster state $|C_4\rangle$, i.e. $F = \max\langle Cl_4|\varrho|Cl_4\rangle$, were the maximum is taken over all square network states ϱ. The fidelity of a state ϱ with the cluster state is given by $F = \frac{1}{16}\sum_{i=0}^{15}\langle S_i\rangle_\varrho$, where $\{S_i\}$ is the stabilizer of $|C_4\rangle$, consisting of $S_0 = 1111$ and further elements given in Supplementary Table I.

The symmetry of $|C_4\rangle$ implies that one can assume the network state ϱ that maximizes the fidelity to admit the same expectation value on operators from the same column, as denoted in the last row of Supplementary Table I.

As explained in the main text, the general idea is to notice that some stabilizers of $|C_4\rangle$ anticommute in the appropriate inflation, and then use the fact that anticommuting operators cannot all have large expectation values for a given state. In the τ-inflation of the square network (see Supplementary Fig. 3), the observable $X_B X_{D'}$ and $Y_A Y_B Z_C Z_D$ anticommute, and since $\tau_{BD'} = \varrho_{BD}$ and $\tau_{ABCD} = \varrho_{ABCD}$ one has

$$\Xi^2 + \Lambda^2 \leq 1. \tag{S19}$$

Secondly, we have Eq. (9) of the main text that we reformulate as

$$\Xi^2 + \Theta^2 + \Sigma^2 \leq 1. \tag{S20}$$

At last, we consider the observables $X_A X_B X_C X_D$ and $Z_A X_B Z_{A'} X_{D'}$ in the inflation τ. However, the latter is not a stabilizer of the four-qubit ring cluster state, but we have $\langle Z_A X_B Z_{A'} X_{D'}\rangle_\tau = \langle Z_A X_B Z_{A'} X_D\rangle_\gamma$. Then, using the fact that for commuting dichotomic measurements, $\langle M_1 M_2\rangle \geq \langle M_1 M_3\rangle + \langle M_2 M_3\rangle - 1$ [1], one gets $\langle Z_A X_B X_D Z_{A'}\rangle_\gamma \geq \langle Z_A X_B Z_C\rangle_\gamma + \langle Z_C X_D Z_{A'}\rangle_\gamma - 1$. Since $X_A X_B X_C X_D$ and $Z_A X_B Z_{A'} X_{D'}$ are anticommuting, from constraints on the marginals, one finally gets

$$2\Theta - 1 \leq \sqrt{1 - \Lambda^2}. \tag{S21}$$

Analogously,

$$2\Sigma - 1 \leq \sqrt{1 - \Lambda^2}, \tag{S22}$$

$$2\Theta - 1 \leq \sqrt{1 - \Omega^2}, \tag{S23}$$

$$2\Sigma - 1 \leq \sqrt{1 - \Omega^2}. \tag{S24}$$

By exploiting all these inequalities as constraints on the maximization of the fidelity, we finally get

$$\begin{aligned} F &= \frac{1}{16}\big(1 + 4\Theta + 4\Lambda + 2\Xi - 4\Sigma + \Omega\big) \\ &\leq 0.737684, \end{aligned} \tag{S25}$$

hence all states with a larger fidelity to the four-qubit ring cluster state cannot be prepared in a square network.

Proof of Observation 1

Here we provide a detailed proof of Observation 1. To do so, we first need to prove the following theorem.

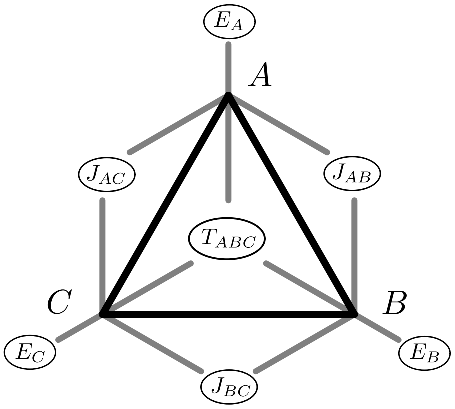

Supplementary Fig. 4: *Illustration of the conditions in Theorem 3.* We consider a triangle in the graph of a graph state. The vertices A, B, C share some neighbourhoods, which are indicated by black ellipses. Note that the graph may contain further vertices, also the vertices in different neighbourhoods may be connected. See the text for further details.

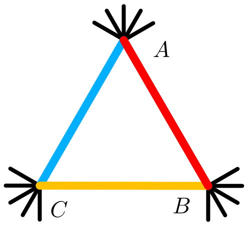

Supplementary Fig. 5: *The bipartite network with complete graph K.* This is the network that should generate the graph state from Supplementary Fig. 4. Note that here the bipartite links of the network are shown, and not the edges of the graph of the graph state.

Theorem 3. *Let $G(V, E)$ be a graph as in Supplementary Fig. 4 with three mutually connected vertices A, B and C and let*

$$T_{ABC} = \mathcal{N}_A \cap \mathcal{N}_B \cap \mathcal{N}_C, \tag{S26}$$
$$J_{AB} = (\mathcal{N}_A \cap \mathcal{N}_B) \setminus T_{ABC}, \tag{S27}$$
$$E_A = \mathcal{N}_A \setminus (\mathcal{N}_B \cup \mathcal{N}_C), \tag{S28}$$

etc., where $\mathcal{N}_X$ is the neighborhood of X ($X = A, B, C$). Then the graph state $|G\rangle$ cannot originate from any network with only bipartite sources, if one of the following conditions is satisfied:

1. *$J_{XY} = J_{XZ} = \emptyset$, where X, Y, Z is a permutation of A, B, C;*
2. *$E_X = E_Y = \emptyset$, where $X \neq Y \in \{A, B, C\}$;*
3. *$E_X = J_{XY} = \emptyset$, where $X \neq Y \in \{A, B, C\}$.*

Proof. We only need to show that the graph state $|G\rangle$ cannot be generated in the network with the complete graph K as shown in Supplementary Fig. 5, where the number of vertices is the same than in G.

We start our discussion with the inflation γ as in Supplementary Fig. 6. Let us denote

$$g_A = X_A Z_{\mathcal{N}_A}, \quad g_B = X_B Z_{\mathcal{N}_B}, \quad g_C = X_C Z_{\mathcal{N}_C}, \tag{S29}$$

where $\mathcal{N}_A$ is the neighborhood of the vertex A in the graph G. Then

$$g_A g_B = Y_A Y_B Z_{R_{AB}}, \tag{S30}$$
$$g_A g_C = Y_A Y_C Z_{R_{AC}}, \tag{S31}$$
$$g_B g_C = Y_B Y_C Z_{R_{BC}}, \tag{S32}$$

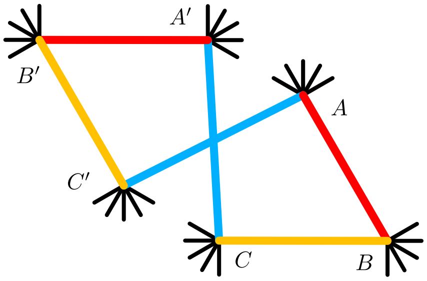

Supplementary Fig. 6: *The network for the inflation* γ. This is a two-copy inflation, where only the links between AC and $A'C'$ are rewired.

where $R_{AB} = E_A \cup E_B \cup J_{AC} \cup J_{BC}$, and analoguously for R_{AC} and R_{BC}. Since $g_A g_C = (g_A g_B)(g_B g_C)$, we can apply the usual argument from the GHZ state to conclude that

$$\langle Y_A Y_C Z_{R_{AC}} \rangle_\gamma \geq \langle Y_A Y_B Z_{R_{AB}} \rangle_\gamma + \langle Y_B Y_C Z_{R_{BC}} \rangle_\gamma - 1. \tag{S33}$$

By comparing the marginals of the states γ and ϱ, we have

$$\langle Y_A Y_B Z_{R_{AB}} \rangle_\gamma = \langle Y_A Y_B Z_{R_{AB}} \rangle_\varrho, \tag{S34}$$

$$\langle Y_B Y_C Z_{R_{BC}} \rangle_\gamma = \langle Y_B Y_C Z_{R_{BC}} \rangle_\varrho, \tag{S35}$$

since $A, C \notin R_{AB} \cup R_{BC}$. In the following, we will also use the notation

$$\mathcal{R} = R_{AC} = E_A \cup E_C \cup J_{AB} \cup J_{BC} \tag{S36}$$

in order to avoid a plethora of indices.

Then, we consider the inflation η shown in Supplementary Fig. 7. This is constructed as follows. First, one has two disconnected complete graphs, K and K'. Then, one takes the subset $\mathcal{R}$ as a subgraph of K and rewires all connections from vertices in $\mathcal{R}$ to A to A'. Similarly, one takes the subset $\mathcal{R}'$ as a subgraph of K' and rewires all connections from vertices in $\mathcal{R}'$ to A' to A.

By comparing the marginals of γ and η, this time we have

$$\langle Y_A Y_C Z_{\mathcal{R}} \rangle_\gamma = \langle Y_{A'} Y_C Z_{\mathcal{R}} \rangle_\eta. \tag{S37}$$

With this reasoning, we have established that the correlation $\langle Y_{A'} Y_C Z_{\mathcal{R}} \rangle_\eta$ is large in η, if the original state ϱ is close to the graph state. Now we have to identify another anticommuting observables in η with large expectation value in order to arrive at a contradiction.

A natural first candidate is the stabilizing operator

$$g_B = X_B Z_A Z_C Z_{\mathfrak{R}}. \tag{S38}$$

with $\mathfrak{R} = E_B \cup J_{AB} \cup J_{BC} \cup T_{ABC}$ of the graph state. This, however, cannot always be identified with some observable in the inflation η. Still, if

$$\mathcal{R} \cap \mathfrak{R} = \emptyset \Leftrightarrow J_{AB} = J_{BC} = \emptyset \tag{S39}$$

the observable g_B is not affected by the rewiring in η, we have $\langle g_B \rangle_\varrho = \langle g_B \rangle_\eta$. Moreover, in η the observables g_B and $Y_A' Y_C Z_{\mathcal{R}}$ anticommute. So, in this case we have

$$\langle Y_A Y_C Z_{\mathcal{R}} \rangle_\gamma^2 + \langle g_B \rangle_\eta^2 \leq 1. \tag{S40}$$

and for the original ϱ we arrive at the condition (assuming $\langle Y_A Y_B Z_{R_{AB}} \rangle_\varrho + \langle Y_B Y_C Z_{R_{BC}} \rangle_\varrho - 1 \geq 0$, as in Eq. (6) in the main text)

$$(\langle Y_A Y_B Z_{R_{AB}} \rangle_\varrho + \langle Y_B Y_C Z_{R_{BC}} \rangle_\varrho - 1)^2 + \langle g_B \rangle_\varrho^2 \leq 1 \tag{S41}$$

for states that can be prepared in the network.

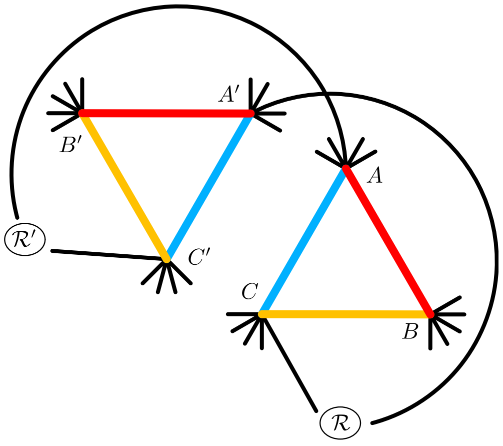

Supplementary Fig. 7: *The network for the inflation η.* This is constructed as follows. First, one has two disconnected complete graphs, K and K'. Then, one takes the subset $\mathcal{R}$ as a subgraph of K and rewires all connections from vertices in $\mathcal{R}$ to A to A'. Similarly, one takes the subset $\mathcal{R}'$ as a subgraph of K' and rewires all connections from vertices in $\mathcal{R}'$ to A' to A.

Furthermore, in the case that $E_A = E_C = \emptyset$ and by making use of the operator

$$g_A g_B g_C = X_A X_B X_C Z_{E_B \cup T_{ABC}}, \tag{S42}$$

we arrive at a similar condition on ϱ that is also violated by the graph state $|G\rangle$.

Finally, if $E_A = J_{AB} = \emptyset$, one can make use of the operator

$$g_A = X_A Z_B Z_C Z_{J_{AC} \cup T_{ABC}}, \tag{S43}$$

in order to arrive at a condition on ϱ that is not satisfied by the graph state $|G\rangle$.

By permuting A, B, C in the above argument, we finish our proof. □

In the following, we identify some basic situations where the Theorem 3 can be applied. First, we show that the conditions of Theorem 3 are met, if there is one vertex with a small degree.

Corollary 4. *Let G be connected graph with no less than three vertices. If its minimal degree is no more than three, the graph state $|G\rangle$ cannot be generated by any network with bipartite sources.*

Proof. The proof will be done successively for minimal degree one, two and three.

Let v be a vertex whose degree is one and let w be the vertex connected to v. Since G is a connected graph with no less than three vertices,

$$\mathcal{N}_w \setminus \{v\} \neq \emptyset, \tag{S44}$$

where $\mathcal{N}_w$ is the neighbourhood of w. If we apply local complementation on the vertex w, we obtain a new graph G', where

$$u \sim v, \quad \forall u \in \mathcal{N}_w \setminus \{v\}, \tag{S45}$$

where $u \sim v$ means that the vertices u, v are connected.

By setting

$$B = w, \quad A = v, \quad C = u_0, \tag{S46}$$

where u_0 is an arbitrary vertex in $\mathcal{N}_w \setminus \{v\}$, we see that

$$\begin{aligned} \mathcal{N}_B \setminus \{A, C\} &= T_{ABC} \cup J_{AB}, \\ A \sim B, \quad A \sim C, &\quad B \sim C. \end{aligned} \tag{S47}$$

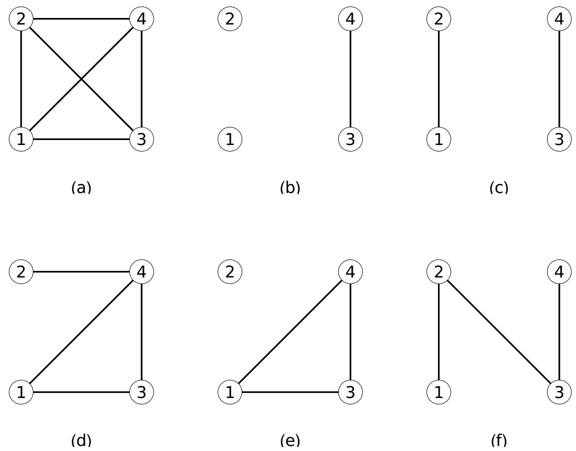

Supplementary Fig. 8: The six inequivalent graphs with four vertices up to permutations and complementation.

Hence, $E_B = J_{BC} = \emptyset$, which implies $|G'\rangle$ cannot be from any network with only bipartite sources. Since $|G\rangle$ is equivalent to $|G'\rangle$ up to a local unitary transformation, we come to the same conclusion for $|G\rangle$.

Now, let us consider graphs with minimal degree equal to two, and let v be a vertex with degree two, and w and u be the two vertices connected to v. If $w \not\sim u$, we can apply a local complementation on v to connect them. Hence, we can assume $w \sim u$ without loss of generality. By setting

$$A = w, \quad B = v, \quad C = u, \tag{S48}$$

we have

$$E_B = J_{AB} = J_{BC} = T_{ABC} = \emptyset, \tag{S49}$$

which leads to the desired conclusion.

Lastly, let v be a vertex with degree three and let w, u and t be the three vertices connected to v. Since we can apply local complementation on v, without loss of generality, we can assume that there are at least two edges among w, u and t, more specifically, $w \sim u$ and $w \sim t$. Let us take

$$A = w, \quad B = v, \quad C = u, \tag{S50}$$

hence we see that

$$E_B = \emptyset, \quad J_{AB} = \emptyset \quad \text{or} \quad J_{BC} = \emptyset. \tag{S51}$$

This implies that the graph state $|G\rangle$ with a vertex of degree three cannot be from any network with only bipartite sources. □

Under certain conditions, we can also exclude a network structure for graphs with minimal degree four:

Corollary 5. *Let G be a graph that has a vertex v of degree four. If the induced subgraph on the neighborhood $\mathcal{N}_v$ is not a line graph, then $|G\rangle$ cannot be generated in any network with bipartite sources.*

Proof. As shown in Supplementary Fig. 8, there are six inequivalent graphs with four vertices up to permutation and complementation. In case (a), we can set $B = v, A = u_1, C = u_2$, then

$$T_{ABC} = \{u_3, u_4\}, \quad E_B = J_{AB} = J_{BC} = \emptyset. \tag{S52}$$

In case (b) and (c), we can set $B = v, A = u_3, C = u_4$, then

$$E_B = \{u_1, u_2\}, \quad J_{AB} = J_{BC} = T_{ABC} = \emptyset. \tag{S53}$$

In case (d) and (e), we can set $B = v, A = u_1, C = u_3$, then

$$E_B = \{u_2\}, \quad T_{ABC} = \{u_4\}, \quad J_{AB} = J_{BC} = \emptyset. \tag{S54}$$

In all the above cases, Theorem 3 implies that the graph state $|G\rangle$ cannot be from any network with only bipartite sources. In case (f), the neighbourhood $\mathcal{N}_v$ of v is a line graph whose complementation is also a line graph. □

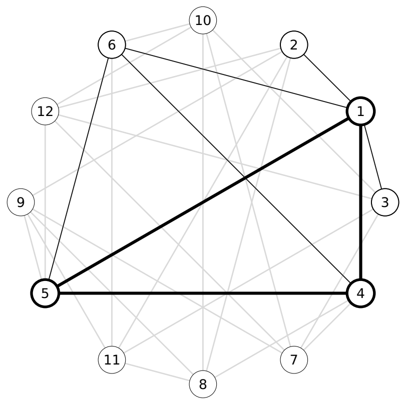

Supplementary Fig. 9: The graph G_{d5} with twelve vertices, where the minimal degree is no less than five whatever local complementation is applied.

Having established these results, we can discuss graphs with a small number of vertices. Here. previous works have established a classification of all small graphs with respect to equivalence classes under local complementation. In detail, this classification has been achieved for up to seven vertices in Ref. [10], for eight vertices in Ref. [14] and for nine to twelve vertices in Ref. [15]. These required numerical techniques are advanced, as, for instance, for twelve qubits already 1 274 068 different equivalence classes under local complementation exist. We can use this classification now, and apply our result on it to obtain:

Theorem 6. *No graph state with up to twelve vertices can originate from a network with only bipartite sources.*

Proof. Using the tables in Ref. [15] one can directly check that except the graph G_{d5} in Supplementary Fig. 9, all graphs with no more than twelve vertices, up to isomorphism and local complementation, satisfy at least one condition in Corollary 4 and 5.

For the graph G_{d5}, the minimal degree is no less than 5 whatever local complementation is applied. However, if we set

$$B = v_1, \quad A = v_4, \quad C = v_5, \tag{S55}$$

then

$$E_B = \{v_2, v_3\}, \quad T_{ABC} = \{v_6\}. \tag{S56}$$

Thus, $J_{AB} = J_{BC} = \emptyset$, which implies that the graph state $|G_{d5}\rangle$ cannot originate from a network with only bipartite sources.

All in all, no graph state with more than twelve vertices can originate from a network with only bipartite sources. □

The statements in Observation 1 concerning the two- or three-dimensional cluster states follow directly from Corollary 5 (in the 2D case) or the application of a local complementation and Theorem 3.

Networks with tripartite sources

Let us finally discuss two examples of networks with tripartite sources. First, one may expand the method used in the main text to exclude the GHZ state of the set of network states. Consider the fully connected four-partite graph state, which is locally equivalent to the four-partite GHZ state and let us show that it cannot have originated from the four partite network with four tripartite sources, illustrated in Supplementary Fig. 10. Consider the ν- and τ-inflations of that network, depicted in Supplementary Fig. 11. From $\langle Y_i Y_k\rangle_\nu \geq \langle Y_i Y_j\rangle_\nu + \langle Y_j Y_k\rangle_\nu - 1$ and the marginal equalities, one gets $\langle Y_A Y_{A'}\rangle_\tau \geq \langle Y_A Y_B\rangle_\varrho + \langle Y_B Y_C\rangle_\varrho + \langle Y_C Y_D\rangle_\varrho + \langle Y_D Y_A\rangle_\varrho - 3$. Then, from an anticommuting relation in the τ-inflation and marginal equalities, one gets $\langle Y_A Y_{A'}\rangle_\tau^2 + \langle X_A Z_B Z_C Z_D\rangle_\varrho^2 \leq 1$. Combining those two equations, one gets that a fidelity higher than 0.923 to the the fully connected graph state cannot be achieved by a four-partite network state.

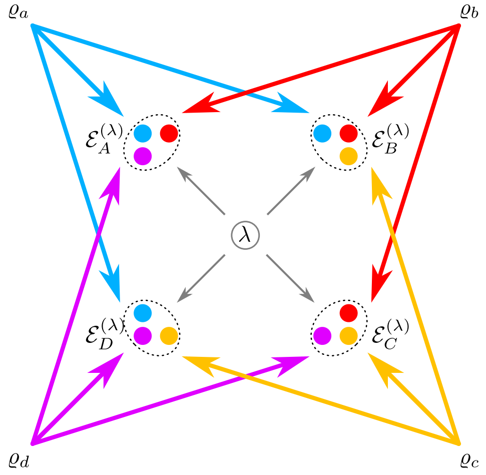

Supplementary Fig. 10: Square network with four tripartite sources.

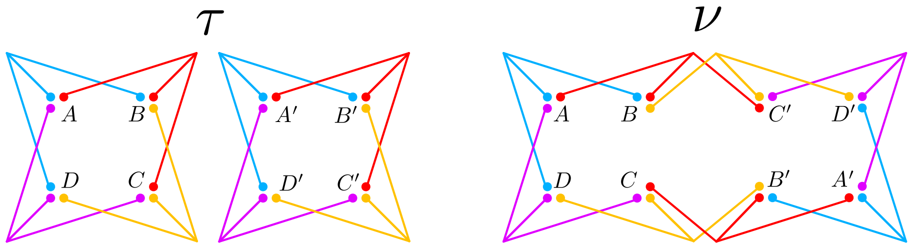

Supplementary Fig. 11: The τ- and ν-inflations of the square network with tripartite sources.

Second, consider the graph state $|G\rangle$ whose graph is represented in Supplementary Fig. 12 (note that it is equivalent under local complementation to the fully connected graph state with the edge between A and D missing). Now, consider a six-partite network with six tripartite sources, connected as in Supplementary Fig. 13. One can show that the observables $X_A X_{D''}$, $Z_A X_B Z_D$ and $Y_A Y_{E'} X_D$ anticommute in the ξ-inflation of the network (see Supplementary Fig. 14) and thus get to an inequality for any state ϱ in the considered network, $\langle X_A X_D\rangle_\varrho^2 + \langle Z_A X_B Z_D\rangle_\varrho^2 + \langle Y_A Y_E X_D\rangle_\varrho^2 \leq 1$, where all three observables are in the stabilizer of $|G\rangle$. Seven other inequalities of that type can be derived, and using them one gets that no state with a fidelity to $|G\rangle$ higher that 0.949 can be prepared in the quantum network of Supplementary Fig. 13.

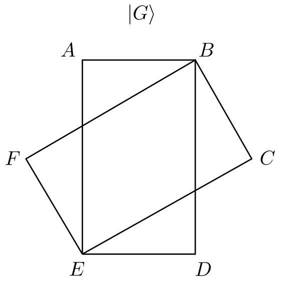

Supplementary Fig. 12: A six-partite graph state $|G\rangle$.

From these two examples, we see that the anticommuting relation method also holds for networks with more then bipartite sources. However, it remains an open question whether it leads to general results similar to those of Observation 1.

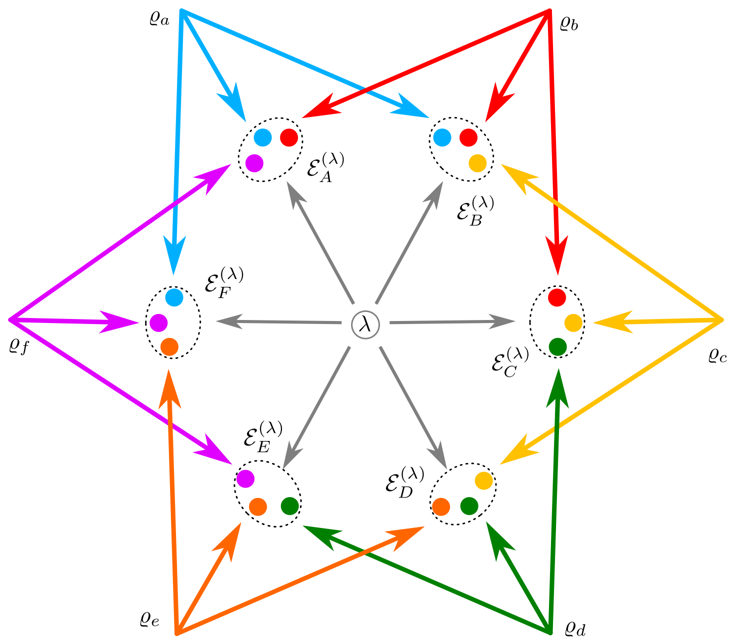

Supplementary Fig. 13: Hexagon network with six tripartite sources.

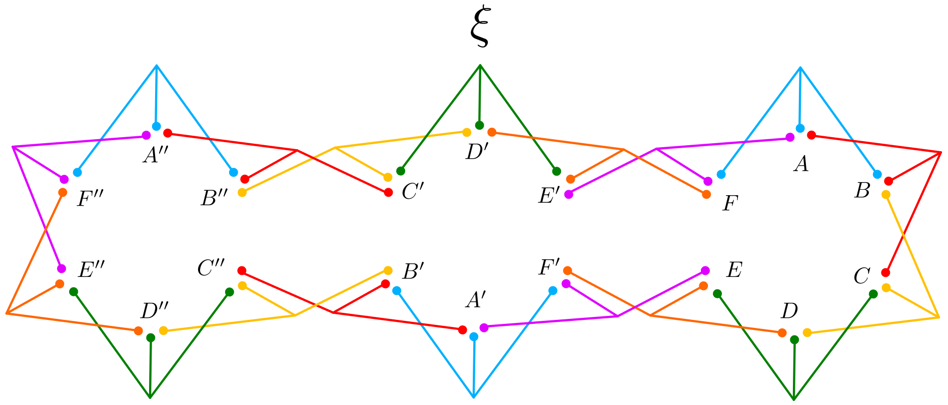

Supplementary Fig. 14: The ξ-inflation of the hexagon network with tripartite sources.

SUPPLEMENTARY NOTE 4: PERMUTATIONALLY SYMMETRIC STATES

Before proving our main results, let us give some definitions. As introduced in the main text, we define N-partite permutationally symmetric (bosonic) states as states that satisfy $\Pi^+_{ij}\varrho\Pi^+_{ij} = \varrho$ for all $i, j \in \{1, \dots, N\}$ with $2\Pi^+_{ij} = \mathbb{1} + F_{ij}$ and F_{ij} being the flip operator that exchanges parties i and j. We can also introduce fermionic states that are antisymmetric for a pair of parties $\{ij\}$, i.e. $\Pi^-_{ij}\varrho\Pi^-_{ij} = \varrho$, with $2\Pi^-_{ij} = \mathbb{1} - F_{ij}$. For our discussion we need several basic facts. We stress that the following Lemma 7 and 9 are well known [16–18], while Lemma 8 is a simple technical statement.

Lemma 7. *Let $\varrho = \sum_k p_k|\psi_k\rangle\langle\psi_k|$ be a multipartite state and let Π be a projector such that $\Pi\varrho\Pi = \varrho$. Then $\Pi|\psi_k\rangle = |\psi_k\rangle$ for all k.*

Proof. One has

$$\begin{aligned} 1 = \mathrm{Tr}(\varrho) =& \mathrm{Tr}(\Pi\varrho\Pi) \\ =& \sum_k p_k\langle\psi_k|\Pi|\psi_k\rangle. \end{aligned} \tag{S57}$$

So $\langle\psi_k|\Pi|\psi_k\rangle = 1$, and since Π is a projector, $\Pi|\psi_k\rangle = |\psi_k\rangle$, for all k. □

This holds in particular for $\Pi = \Pi_{ij}^{\pm}$. As a second lemma, we have

Lemma 8. *If the reduced state on AB of some state is symmetric or antisymmetric under the exchange of parties A and B, then the global state also is.*

Proof. Let $\varrho = \sum_k p_k |\psi_k\rangle\langle\psi_k|$ be the state of some tripartite system ABC. Let us prove that if $F_{AB}\left(\mathrm{Tr}_C(\varrho)\right) = \pm\mathrm{Tr}_C(\varrho)$, then $(F_{AB} \otimes \mathbb{1})\varrho = \pm\varrho$. If one considers the Schmidt decomposition of $|\psi_k\rangle$ wrt the bipartition $AB|C$, one has

$$\varrho = \sum_k p_k \sum_{i,j} s_{k,i} s_{k,j}^* |\phi_{k,i}^{AB}\rangle\langle\phi_{k,j}^{AB}| \otimes |\chi_{k,i}^{C}\rangle\langle\chi_{k,j}^{C}|. \tag{S58}$$

From that, $\varrho_{AB} = \sum_k p_k \sum_i |s_{k,i}|^2 |\phi_{k,i}^{AB}\rangle\langle\phi_{k,i}^{AB}|$ and since it is a permutationally symmetric (respectively antisymmetric) state, from Lemma 7 all states in its decomposition also are and thus $(F_{AB} \otimes \mathbb{1})\varrho = \pm\varrho$. □

We note that for both those lemma, the converse is trivial. Finally, we have:

Lemma 9. *(a) A N-partite symmetric state ϱ_s is either genuinely N-partite entangled or fully separable. (b) A N-partite antisymmetric state is always N-partite entangled.*

Proof. Due to Lemma 7 we only need to consider pure states. Let $|\Psi\rangle$ be a N-partite (anti)symmetric state that is not N-partite entangled, hence it is separable for some bipartition. Without loss of generality, we assume that

$$|\Psi\rangle = |\varphi_{1,\ldots,t}\rangle \otimes |\phi_{t+1,\ldots,N}\rangle. \tag{S59}$$

Thus, by tracing out the first t parties, we have a pure state. The symmetry of $|\Psi\rangle$ implies that the reduced state is pure after tracing out any t parties. This can only be true if $|\varphi_{1,\ldots,t}\rangle, |\phi_{t+1,\ldots,N}\rangle$ are fully separable.

Besides, denote $|ab\ldots c\rangle$ a normalized fully separable antisymmetric state, we have $|ab\ldots c\rangle = -|ba\ldots c\rangle$. This implies that $-1 = \langle ab\ldots c|ba\ldots c\rangle = |\langle a|b\rangle|^2 \geq 0$, hence we arrive at a contradiction. □

We note that the notions of entanglement used in this Lemma are the standard ones for non-symmetric states, as these are the relevant ones for the main text. In principle, for indistinguihable particles one may separate the "formal" entanglement due to the wave function symmetrization from the "physical" entanglement [16].

Now, let us prove the Observation 2 of the main text. For completeneness, we restate it here in the full formulation:
Observation 2'. *Let ϱ be a permutationally symmetric multiparticle state. Then, ϱ can be generated in a quantum network with $N-1$-partite sources if and only if it is fully separable. If ϱ be a permutationally antisymmetric, then it cannot be generated in a network.*

Proof. Let ϱ be a N-partite permutationally (anti)symmetric state. Let us assume that it can be generated in a network of N nodes with some at most $(N-1)$-partite sources. Note that any state that can be generated in a network of N nodes with no N-partite sources, can also be generated in a network of N nodes with N different $(N-1)$-partite sources. Let us denote by ς_i the source used to generate ϱ that distributes parties to all nodes except the ith one.

If we assume that ϱ is a network state, then the inflation η build the following way is a physical state: Consider a network of $2N$ nodes $\{A_i, A_i' : i = 1, \ldots, N\}$ and $2N$ sources $\{\zeta_k, \zeta_k' : k = 1, \ldots, N\}$ that distribute parties to

$$\zeta_k : A_1 \ldots A_{k-1} A_{k+1}' \ldots A_N', \tag{S60}$$
$$\zeta_k' : A_1' \ldots A_{k-1}' A_{k+1} \ldots A_N, \tag{S61}$$

where $\zeta_k = \zeta_k' = \varsigma_k$ for all k. The state η is the network state build with these sources and the same channels on the nodes than ϱ (with some shared randomness). From the inflation technique, we know that for the reduced states

$$\eta_{A_i A_{i+1}} = \eta_{A_i' A_{i+1}'} = \varrho_{A_i A_{i+1}}, \quad \forall i \leq N-1, \tag{S62}$$
$$\eta_{A_1 A_N'} = \eta_{A_1' A_N} = \varrho_{A_1 A_N}. \tag{S63}$$

Since the state ϱ is fully (anti)symmetric, Lemma 8 and Eq. (S62) imply that the state η is also fully (anti)symmetric.

Now, we consider the inflated state τ, whose sources $\{\omega_k, \omega_k' : k = 1, \ldots, N\}$ distribute states to

$$\begin{aligned} \omega_k &: A_1 A_2 A_3 \ldots A_{k-1} A_{k+1} \ldots A_N \\ \omega_k' &: A_1' A_2' A_3' \ldots A_{k-1}' A_{k+1}' \ldots A_N'. \end{aligned} \tag{S64}$$

Again, the local channels and shared randomness are the same than for ϱ. This is the two-copy inflation considered several times in the main text. One has

$$\tau_{A_1\dots A_N} = \tau_{A_1'\dots A_N'} = \varrho. \tag{S65}$$

Moreover,

$$\tau_{A_iA_i'} = \eta_{A_iA_i'} \tag{S66}$$

hence τ is permutationally fully (anti)symmetric under the exchange of all its parties. However, τ is separable wrt the bipartition $A_1\dots A_N|A_1'\dots A_N'$. In the fully symmetric case, this means that τ is fully separable. Therefore ϱ is also fully separable. So, if a network state is permutationally symmetric, it needs to be fully separable. In the fully antisymmetric case, the full separability of τ contradicts with the assumption that τ is fully antisymmetric. So, no network state can be permutationally antisymmetric. □

Finally, we note that cyclic symmetric states may be generated in network scenarios, as already pointed out by Ref. [2]. As an example, we consider three Bell pairs $|\Phi^+\rangle$ as sources in the triangle network, and no channels applied. The global 3×4-partite state is $|\Psi\rangle_{ABC} = |\Phi^+\rangle_{A_2B_1}|\Phi^+\rangle_{B_2C_1}|\Phi^+\rangle_{C_2A_1}$, with $A = A_1A_2$ and so on. With the appropriate reordering of the parties and by mapping $|ij\rangle_X \mapsto |2i+j\rangle_X$ for $X = A, B, C$, one gets

$$|\Psi\rangle_{ABC} = \frac{1}{2\sqrt{2}}\big(|000\rangle + |012\rangle + |120\rangle + |201\rangle + |132\rangle + |321\rangle + |213\rangle + |333\rangle\big), \tag{S67}$$

which is a symmetric state under cyclic permutations of the 3×4-dimensional system ABC.

SUPPLEMENTARY NOTE 5: CERTIFYING NETWORK LINKS

Here, we prove the statement made in the main text, which can be formulated as follows:

Observation 10. *If a state ϱ can be prepared in a network with bipartite sources but without the link AC, then*

$$\langle X_AX_CP_{R_1}\rangle^2 + \langle Y_AY_CP_{R_2}\rangle^2 + \langle Z_AZ_CP_{R_3}\rangle^2 \leq 1. \tag{S68}$$

Here the P_{R_i} are arbitrary observables on disjoint subsets of the other particles, $R_i\cap R_j = \emptyset$. If the state ϱ was indeed prepared in a real quantum network, then violation of this inequality proves that the link AC is working and distributing entanglement.

Proof. Without loss of generality, we assume that

$$R_1 = \{E\}, \quad R_2 = \{B\}, \quad R_3 = \{D\}. \tag{S69}$$

Otherwise, we can prove the result similarly. The disconnected nodes A and C may be connected via some source with the R_i or not, but this is not essential. Then, the graph has a structure as the graph in Supplementary Fig. 15.

We consider a three-copy inflation ξ of this graph, where the observables in Eq. (S68) overlap only in the node A. This inflation is constructed as follows: All links from B to C are rewired from B to C' and all links from D to C are rewired from D to C''. This is shown schematically in Supplementary Fig. 15.

The anticommuting relations imply that

$$\langle X_AX_CP_E\rangle_\xi^2 + \langle Y_AY_{C'}P_B\rangle_\xi^2 + \langle Z_AZ_{C''}P_D\rangle_\xi^2 \leq 1. \tag{S70}$$

By comparing the marginals of ϱ and ξ, we have

$$\langle X_AX_CP_E\rangle_\xi = \langle X_AX_CP_E\rangle_\varrho, \tag{S71}$$
$$\langle Y_AY_CP_B\rangle_\xi = \langle Y_AY_CP_B\rangle_\varrho, \tag{S72}$$
$$\langle Z_AZ_CP_D\rangle_\xi = \langle Z_AZ_CP_D\rangle_\varrho. \tag{S73}$$

By substituting the mean values with state ξ by the ones with ϱ in Eq. (S70), we complete our proof. □

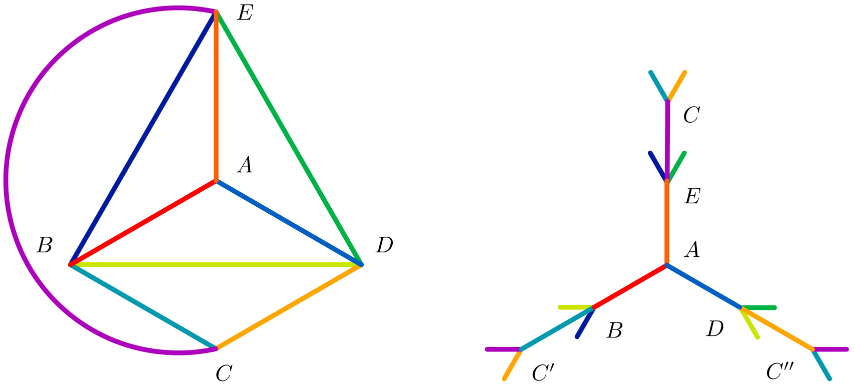

Supplementary Fig. 15: *Left:* A typical graph for a network where the link AC is missing. *Right:* Schematic view of the third order inflation. See text for further details.

Finally, we give a simple example where this criterion can detect the functionality of a link, while a simple concentration on the reduced two-qubit density matrix does not work. Consider the state

$$\varrho = \frac{1}{2}(|s_1\rangle\langle s_1| + |s_2\rangle\langle s_2|), \tag{S74}$$

where

$$|s_1\rangle = (|00\rangle_{AC} + |11\rangle_{AC}) \otimes |00\rangle_{BD}, \tag{S75}$$

$$|s_2\rangle = (|00\rangle_{AC} - |11\rangle_{AC}) \otimes |11\rangle_{BD}. \tag{S76}$$

Here, we want to check whether the link AC works or not. Since the reduced state on AC is separable, we cannot use the criteria which acts only on AC. It is easy to verify that

$$\begin{aligned} &\langle X_A \mathbb{1}_B X_C \mathbb{1}_D\rangle = 0, \\ -&\langle Y_A \mathbb{1}_B Y_C Z_D\rangle = \langle Z_A \mathbb{1}_B Z_C \mathbb{1}_D\rangle = 1, \end{aligned} \tag{S77}$$

which violates Eq. (S70). Hence, our criteria can detect the link AC more effectively.

[1] M. Navascués, E. Wolfe, D. Rosset, and A. Pozas-Kerstjens. Genuine Network Multipartite Entanglement. Phys. Rev. Lett. **125**, 240505 (2020).

[2] M.-X. Luo. New Genuinely Multipartite Entanglement. Adv. Quantum Technol., **4**, 2000123 (2021).

[3] O. Gühne and M. Seevinck. Separability criteria for genuine multiparticle entanglement. New J. Phys. **12**, 053002 (2010).

[4] E. Wolfe, R. W. Spekkens, and T. Fritz. The Inflation Technique for Causal Inference with Latent Variables. J. Causal Inference **7**, 20170020 (2019).

[5] E. Wolfe, A. Pozas-Kerstjens, M. Grinberg, D. Rosset, A. Acín, and M. Navascués. Quantum Inflation: A General Approach to Quantum Causal Compatibility. Phys. Rev. X **11**, 021043 (2021).

[6] J. Åberg, R. Nery, C. Duarte, and R. Chaves. Semidefinite Tests for Quantum Network Topologies. Phys. Rev. Lett. **125**, 110505 (2020).

[7] T. Kraft, C. Spee, X.-D. Yu, and O. Gühne. Characterizing quantum networks: Insights from coherence theory. Phys. Rev. A **103**, 052405 (2021).

[8] N. Gisin, J. D. Bancal, Y. Cai, et al. Constraints on nonlocality in networks from no-signaling and independence. Nat. Commun. **11**, 2378 (2020).

[9] M. Hein, W. Dür, J. Eisert, R. Raussendorf, M. Van den Nest, and H.-J. Briegel, in *Quantum Computers, Algorithms and Chaos*, Proceedings of the International School of Physics "Enrico Fermi," Vol. 162, Varenna, 2005, edited by G. Casati, D. L. Shepelyansky, P. Zoller, and G. Benenti (IOS Press, Amsterdam, 2006). See also arXiv:quant-ph/0602096.

[10] M. Hein, J. Eisert, and H. J. Briegel. Multiparty entanglement in graph states. Phys. Rev. A **69**, 062311 (2004).

[11] D. Gotteman. Stabilizer Codes and Quantum Error Correction. PhD Thesis, Caltech (1997).

[12] K. Audenaert and M. B Plenio. Entanglement on mixed stabilizer states: normal forms and reduction procedures. New J. Phys. **7**, 170 (2005).

[13] M. Van den Nest, J. Dehaene, and B. De Moor. Graphical description of the action of local Clifford transformations on graph states. Phys. Rev. A **69**, 022316 (2004).

[14] A. Cabello, A.J. Lopez-Tarrida, P. Moreno, and J.R. Portillo. Compact set of invariants characterizing graph states of up to eight qubits. Phys. Rev. A **80**, 012102 (2009).
[15] L. E. Danielsen, Database of Entanglement in Graph States (2011), see also `http://www.ii.uib.no/~larsed/entanglement/`.
[16] K. Eckert, J. Schliemann, D. Bruß, and M. Lewenstein. Quantum Correlations in Systems of Indistinguishable Particles. Ann. Phys. **299**, 88 (2002).
[17] G. Tóth and O. Gühne. Entanglement and Permutational Symmetry. Phys. Rev. Lett. **102**, 170503 (2009).
[18] B. Kraus, PhD Thesis, University of Innsbruck (2003).
